# Case Report: Comorbid Hyper-IgD Syndrome and Hidradenitis Suppurativa – A New Syndromic Form of HS? A Report of Two Cases

**DOI:** 10.3389/fimmu.2022.883811

**Published:** 2022-05-26

**Authors:** Philippe Guillem, Dillon Mintoff, Mariam Kabbani, Elie Cogan, Virginie Vlaeminck-Guillem, Agnes Duquesne, Farida Benhadou

**Affiliations:** ^1^Department of Surgery, Clinique du Val d’Ouest, Lyon, France; ^2^ResoVerneuil, Paris, France; ^3^European Hidradenitis Suppurativa Foundation e.V., Dessau, Germany; ^4^Department of Dermatology, Mater Dei Hospital, Msida, Malta; ^5^Department of Pathology, Faculty of Medicine and Surgery, University of Malta, Msida, Malta; ^6^Department of Dermatology, Hôpital Universitaire Erasme, Université Libre de Bruxelles, Brussels, Belgium; ^7^Department of Internal Medicine, Hôpital Universitaire Erasme, Université Libre de Bruxelles, Brussels, Belgium; ^8^Departement of Internal Medecine, Hôpital Delta, CHIREC, Brussels, Belgium; ^9^Université Claude Bernard Lyon 1, INSERM 1052, CNRS 5286, Centre Léon Bérard, Centre de 9 Recherche en Cancérologie de Lyon, Lyon, France; ^10^Centre de Biologie Sud, Hôpital Lyon-Sud, Hospices Civils de Lyon, Pierre-Bénite, France; ^11^Department of Paediatric Nephrology, Rheumatology and Dermatology, Hôpital Femme Mère Enfant, Hospices Civils de Lyon, Bron, France

**Keywords:** hidradenitis suppurativa, mevalonate kinase deficiency, hyper-IgD syndrome, autoinflammatory keratinization disease, autoinflammation

## Abstract

Hidradenitis Suppurativa (HS) is a chronic suppurative disease of the pilosebaceous unit. The current model of HS pathophysiology describes the condition as the product of hyperkeratinisation and inflammation at the hair follicular unit. Environmental factors (such as smoking and obesity), gender, genetic predisposition, and skin dysbiosis are considered the main pathogenic drivers of the disease. Autoinflammatory syndromes associated with HS are rare but may help to highlight the potential roles of autoinflammation and dysregulated innate immune system in HS. Therefore, it is of major relevance to increase the awareness about these diseases in order to improve the understanding of the disease and to optimize the management of the patients. Herein, we report for the first time, to our knowledge, two clinical cases of Hyper-IgD syndrome-associated HS. Hyper-IgD is an autoinflammatory syndrome caused by a mevalonate kinase deficiency (MKD), a key kinase in the sterol and isoprenoid production pathway. We describe the potentially shared pathophysiological mechanisms underpinning comorbid MKD-HS and propose therapeutic options for the management of these patients.

## Introduction

Hidradenitis suppurativa (HS) is a chronic debilitating skin disorder having an estimated overall prevalence of 0.3% (95%CI, 0.2%-0.5%) (1). Patients suffering from HS experience recurrent inflammatory nodules, draining abscesses, and tunnels mainly in, but not limited to intertriginous skin (2). HS is characterized by significant clinical heterogeneity which complicates disease severity stratification and phenotype classification (3). Several HS severity scoring systems exist, of which Hurley staging is the most widely used static score in clinical practice (4). Hurley stage I corresponds to localized disease with single or multiple nodules/abscesses, but lack sinus tracts, this being a defining feature of Hurley stage II. Hurley stage III is the most severe form of HS wherein patients exhibit multiple nodules, abscesses, and sinus tracts involving an entire skin region. In contrast to Hurley staging, the international HS severity score system (IHS4) is a dynamic severity score which assess the inflammatory activity of the disease. The score requires counting individual nodules, abscesses, and draining fistula/sinus tracts in order to classify the disease as mild, moderate, or severe (5). The heterogeneity of the clinical presentation results from complex and incompletely understood inflammatory mechanisms (6). Epidermal hyperplasia of the infundibular portion of the hair follicle is an early pathophysiological event in the development of HS. This process is under the influence of various proinflammatory cytokines including interleukin (IL)-23, IL-12 and IL-17 (7). Disease progression is potentiated by a predominantly neutrophilic inflammatory response involving IL-1, tumor necrosis factor (TNF)- α, IL-17 among other cytokines (6). In addition, upregulation of IL-17, IL-1β, IL-36, and caspase-1 suggests that autoinflammation may play a role in the pathogenesis of the disease (7). Interestingly, rare autoinflammatory syndromes have HS as a key cutaneous manifestation (8). Patients with syndromic HS typically suffer from severe forms of HS with unusual skin location and signs of systemic inflammation, and resistance to conventional treatments is often reported (9). Treatment of syndromic HS is challenging and should be personalized (10). Mounting evidence suggests that HS is a subtype of autoinflammatory keratinization diseases (AiKD) (11). AiKDs are characterized by autoinflammation involving the epidermis and upper dermis which leads to hyperkeratosis as observed in psoriasis, pityriasis rubra pilaris, and keratosis lichenoides chronica. The primary theory supporting HS as an AiKD is based on the fact that identified, potentially causative HS genes [including *Mevalonate kinase (MVK)* (6)], lead to keratinization, autoinflammation, or both (12). HS patients having comorbid monogenic inflammatory syndromes can provide insight into potential convergent or shared inflammatory pathways, thereby piecing together the HS pathophysiological puzzle in order to improve the therapeutic management of HS. Herein we report two patients suffering from comorbid HS and mevalonate kinase deficiency (MKD) associated with Hyper IgD syndrome (HIDS).

## Reports

### Case 1

**Figure 1:**
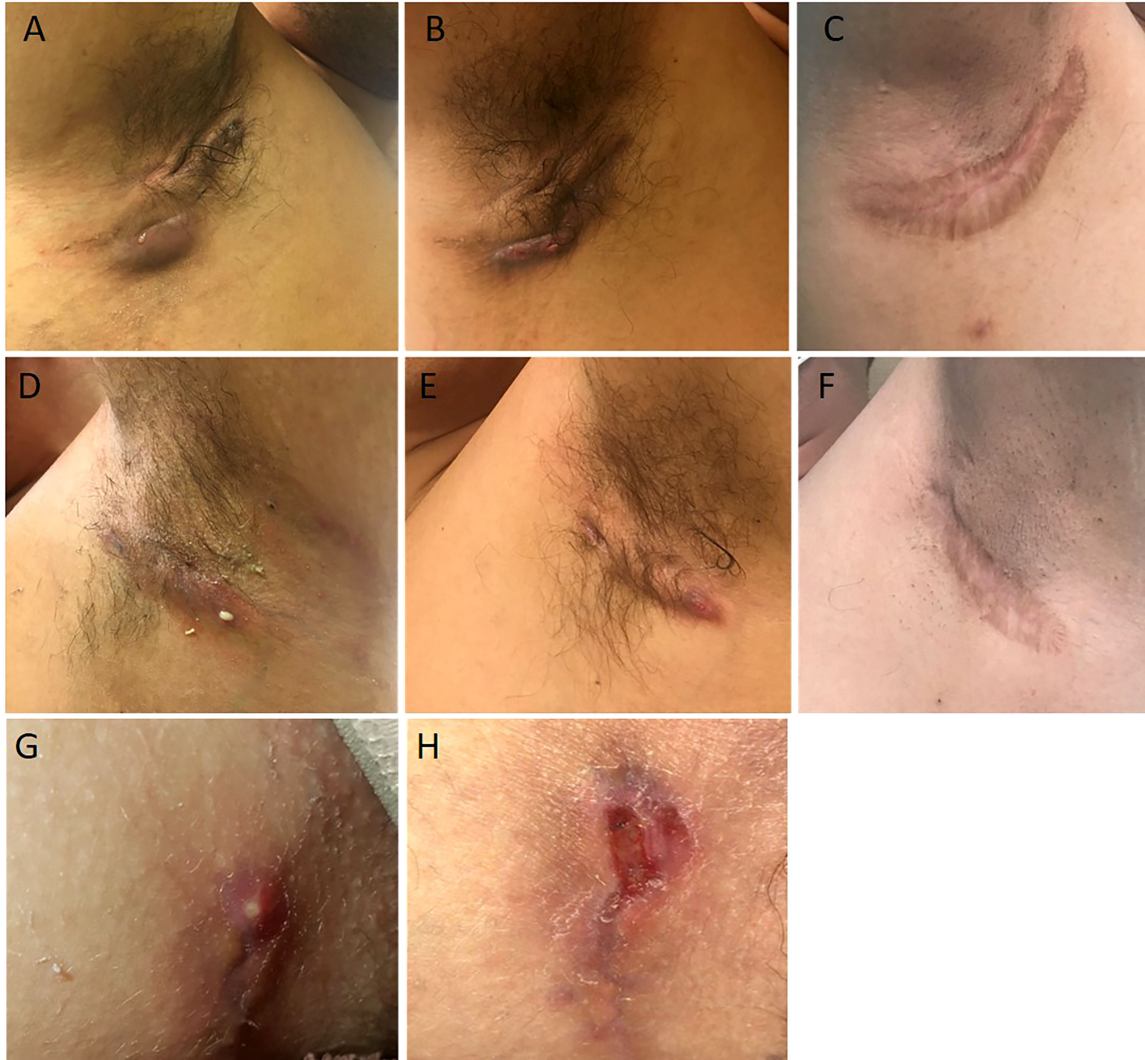
Illustrations of the case reports 1 **(A–F)** and 2 **(G, H)**. Abscesses and draining fistulae of the right armpit at baseline **(A)**, decrease of inflammation and suppuration 3 months after initiation of adalimumab **(B)**, wide excision of remaining non draining fistulae 1 year after initiation of adalimumab **(C)**. Draining fistulae of the left armpit at baseline **(D)**, decrease of inflammation and suppuration 3 months after initiation of adalimumab **(E)**, wide excision of remaining non draining fistulae 1 year after initiation of adalimumab **(C)**. Initial Hurley 2 lesion of case report 2 **(G)**. Recurrence after limited excision of the lesion and direct suture **(H)**.

The proband, a 34-year-old-man of Moroccan ethnicity, presented to a dermatology tertiary center with a 15-year history of HS Hurley 3 involving the groin, axillae, and perianal region. Additionally, the patient experienced recurrent episodes of pyrexia reaching 41°C persisting for up to a week. The febrile episodes recurred on an eight-weekly basis and were frequently accompanied by systemic symptoms including abdominal pain, myalgia, and arthralgia. The patient’s sister suffered from HS Hurley 1 involving the buttocks and axillae. Additionally, the patient’s brother suffered from Crohn’s disease but did not manifest HS. Physical examination of the proband revealed inflammatory nodules, abscesses, and draining fistulae in his axillae. The patient had not responded to treatment with standard antibiotic therapy, namely tetracycline and clindamycin-rifampicin combination, or to surgical incision and drainage of abscesses in the perianal region. In view of the history and clinical findings, an underlying periodic fever syndrome was suspected. Whole exome sequencing (WES) of the proband revealed him to be homozygous for the *MVK* (NM_001114185.3):c.1129G>A (p.Val377Ile) variant. This variant is classified pathogenic according to ACMG (7). The predicted effects of the variant are summarized in **Table S1**. The patient’s siblings refused genetic testing. A diagnosis of HIDS was suspected and subsequently confirmed upon finding elevated levels of serum IgD. Recurrent febrile episodes necessitated treatment with multiple courses of oral corticosteroids. Colchicine and simvastatin were ineffective at maintaining disease remission. In view of comorbid HS and first-line treatment failure, the patient was prescribed the tumor necrosis factor (TNF)-α blocker adalimumab, starting with a loading dose of 160mg followed by 80mg after two weeks and subsequently 40mg every week. At three months, the patient experienced significant improvement of inflammatory nodules and abscesses (**Figures 1A, B, D, E**); however, the draining tunnels persisted. The patient also experienced complete remission of febrile episodes. Levels of C-Reactive Protein (CRP) decreased from 62 at baseline to 8 mg/l (normal range: < 10mg/l). Quiescence of both HS and HIDS provided a window of opportunity allowing for wide local excision of the axillae to be performed (**Figures 1C, F**). Maintenance therapy with adalimumab retained the patient in complete remission from both HS and HIDS for 13 months. This encouraged the patient to stop taking adalimumab of his own accord. Notwithstanding, he has not experienced fever or HS lesions at follow up three years later. **Table 1** summarizes the key data of case 1.

**Table 1:** Characteristics of the probands. Key data of case 1 and case 2.

	Proband 1	Proband 2
**Age at presentation (years)**	34	15
**Gender**	Male	Female
**MKD medical history** Age at symptom onset (years) Symptoms Previous unsuccessful therapies Genetic variants	8 Fever attacks/8 weeks, abdominal pain, arthralgia, myalgia Oral corticosteroids, colchicine and simvastatin Homozygous for the MVK (NM_001114185.3):c.1129G>A (p.Val377Ile) variant	5 Fever attacks/8 weeks, abdominal pain, arthralgia Oral corticosteroids, anakinra and canakinumab Heterozygote for MVK variants; 117 NM_001114185.3:c.1129G>A (p.Val377Ile) /NM_001114185.3:c.612C>G(p.Asp204Glu)
**HS medical history** Age at symptom onset (years) HS location Hurley stage at examination IHS4 at examination Previous unscuccessful therapies	19 Initially localized in perianal folds and then extended to axillary and inguinal folds 3 18 (=Severe HS) Several lines of oral antibiotics including tetracycline and clindamycin-rifampycin and local incision	14 Initially localized in the inguinal folds and then extended to inner thighs and labia majora 2 14 (=Severe HS) Several lines of oral antibiotics and local excision
**Familial history**	Sister : HS Hurley 1 Brother : Crohn disease	None
**Therapeutic strategies used successfully to control HS and MKD**	Adalimumab and wide surgical excision	Adalimumab +canakinumab+ wide surgical excision

Proband 1 corresponds to the case report 1 and Proband 2 to the case report 2. MKD, Mevalonate kinase deficiency; HS, Hidradenitis suppurativa.

### Case 2

A 15-year-old female presented with a long-standing history of HIDS characterized by a history of recurrent febrile episodes, abdominal pain, and arthralgia occurring every 8 weeks. Targeted sequencing had revealed the patient to be a compound heterozygote for *MVK* variants*;* 117 NM_001114185.3:c.1129G>A (p.Val377Ile) and NM_001114185.3:c.612C>G(p.Asp204Glu). This variant is classified as likely pathogenic according to ACMG criteria. The predicted effects of the variants are summarized in **Table S1**. The patient’s serum IgD level was five-fold the upper limit of normal. The CRP at baseline was 25mg/l and we found both elevated urinary mevalonic acid (55.7 mmol/mol creatinine; Normal ranges: <1.3) and reduced MVK enzyme activity in lymphocytes (0.2 µkat/kg prot; normal range: 2.8 – 6.8).Treatment with oral steroids and anakinra (an IL-1α and β inhibitor) at age 6 was ineffective at controlling febrile attacks. For this reason, anakinra was replaced by 6-weekly injections of canakinumab, a specific IL-1β inhibitor. Nevertheless, the febrile episodes persisted and at 14 years of age the patient started developing recurrent abscesses and draining fistulae in her groin, inner thighs, and the major labia consistent with a diagnosis of Hurley 2 HS. Several courses of oral antibiotics and limited excisions procedures were unsuccessfully performed until HS diagnosis (**Figures 1G, H**). For this reason, adalimumab (40mg every other week) was added on to canakinumab. This combination therapy resulted in a significant decrease in HS symptoms as well as better control of febrile attacks allowing a dose spacing of canakinumab from 6 weeks to 9-10 weeks. The patient underwent wide excision of a Hurley 2 lesion in the groin with a stable disease. **Table 1** summarizes the key data of case 2.

## Discussion

Autoinflammatory diseases (AIDs) are disorders characterized by recurrent episodes of inflammation resulting in a broad range of symptoms including recurrent fevers, as well as musculoskeletal and digestive manifestations. In view of the highly heterogenous clinical manifestation, genotype-phenotype correlations are difficult to establish. Monogenic HS has been observed in a minority of patients who harbor gamma-secretase-encoding genes mutations (13) or when associated with others AIDs within syndromic disorders such as PASH, PAPASH, and other related syndromes (14). Although detected in patients with PASH, the *MVK* variants described showed no relevant nucleotide substitution (8).

Patients with concomitant HIDS-HS have not yet been described. Less than 200 patients with HIDS have been reported in case series worldwide. Therefore, it is difficult to ascertain whether the occurrence of comorbid HS-HIDS is serendipitous or the repercussion of shared, underlying inflammatory processes (15) and thus be considered as yet another example of syndromic (AID-associated) HS.

In clinical practice, HIDS is symptomatic in early childhood while HS usually manifests later in life, as observed in our two patients. This suggests that this association has to be assessed in HIDS patients rather than in HS patients. Since HS remains largely unrecognized and underdiagnosed, with a significant global diagnostic delay (9), the condition may remain undiagnosed in patients suffering from comorbid inflammatory conditions such as HIDS. As an example, Ekinci et al. reported the co-occurrence of HIDS and recurrent perianal abscess in an 18-month-old boy which was illustrated in their case report with picture of the abscess highly compatible with a HS lesion (10). Recently, in supplemental data of a large series of published cases of patients harboring *MVK* variants, Boursier et al. provided data about mucocutaneous manifestations in 180 patients (11). Out of them, 7 (4%) presented with HIDS-associated folliculitis. One should consider whether the diagnosis of folliculitis actually represents unrecognized HS, since at least one of these seven cases is reported as suffering from severe acne rather than folliculitis in the original publication (12).

**Figure 2:**
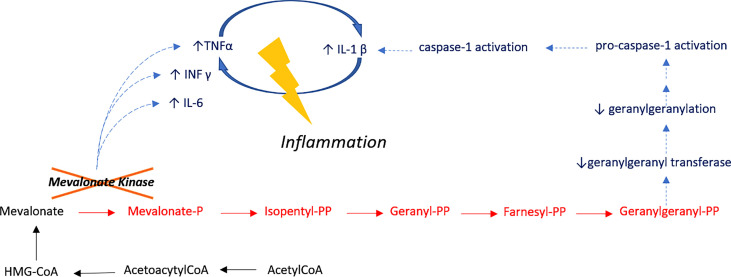
Inflammatory Pathway in HIDS. Mevalonate kinase catalyzes the phosphorylation of mevalonic acid a key enzyme of the mevalonate pathway I, involved in cholesterol metabolism. Treg cell function is dependent on mevalonate pathway-orchestrated lipid oxidation. Defective cholesterol metabolism has also been shown to induce human B cell regulatory program by metabolic priming. As such, B cells of patients who are deficient in MVK are functionally impaired and have a reduced capacity to produce IL-10 upon stimulation. Impaired production of IL-10 results in the unabated induction of CD4 T cells by Th1. IL-10 also suppresses inflammation through paracrine and autocrine mechanisms, controlling the differentiation of B-cells into IgM or IgG secreting plasmablasts. Unstimulated peripheral blood mononuclear cells from HIDS patients produce increased amounts of pro-inflammatory mediators including cytokines, such as IL-1, IL-6 and TNF-α.

Our two patients carry the most frequent HIDS-associated *MVK* variant (p.Val377Ile), and it remains unclear why these patients manifested HS whilst other patients bearing the same variant do not. The allele frequency of the single nucleotide variant is 0.001578. Both patients had a moderate form of HIDS and it is unclear if HS-associated HIDS has other clinical characteristics. Both patients being described were non-smokers and lean, suggesting that inflammation was the key driver in pathogenesis (rather than environmental factors). Despite turning down genetic assessment, the patient’s sibling discussed in case one suffered from Crohn’s disease, which is associated with HS (PMC8130070). In both cases, we observed a lack of response to standard HS treatment modalities, which albeit a frequent occurrence, may be more significant in patients with underlying genetic variation and can be considered a negative predictive biomarker (9). Of note, several inactivating mutations have also been reported in genes located downstream to the *MVK* gene in the isoprenoid synthesis pathway. HS screening should also be performed in such patients to identify which *MVK*-related disorders are preferentially associated with HS. HIDS belongs to the AID-subgroup of ‘inflammasomopathies’ (13) as HS and all syndromic HS-associated AIDs. HS-associated monogenic AIDs are indeed related to mutations in genes involved in the regulation of inflammasome, a multiprotein complex that regulates the biosynthesis of IL-1β and IL-18 (14). The role of IL-1β in systemic inflammatory diseases is crucial and should be explored in HS. The therapeutic value of IL-1 inhibitors for the treatment of HS has been investigated with diverse results (15). Apart from TNF-α and IL-1, other, potentially pathogenic inflammatory drives include IL-36α, IL-36β and IL-36γ which are overexpressed in HS skin lesions (**Figure 2**) and may be considered as future therapeutic targets. The role of MVK may not be limited to inflammation, but also hyperkeratinization (a key pathophysiological process in HS) as is the case with porokeratosis considered an AiKD (5) which is driven by hyperplasia of infundibular keratinocytes of the pilosebaceous unit.

Future descriptions of HIDS-HS associations and syndromic HS are likely to lead to both a better understanding of the underlying pathophysiological mechanisms and a better adaptation of diverse biotherapy-based therapeutic strategies. Therefore, we suggest that these previously unreported associations of HS and MKD advocate for the existence of a new syndromic HS wherein the standard HS treatments fail to provide a satisfactory response. The success of anti-TNF-α therapy with or without the co-administration of anti-IL-1 agents supports the pivotal role of these cytokines in the pathogenesis of this novel AID whose specific traits might be further elucidated by future reports of co-morbid HIDS-HS syndrome.

## Data Availability Statement

The raw data supporting the conclusions of this article will be made available by the authors, without undue reservation.

## Ethics Statement

Ethical review and approval was not required for the study on human participants in accordance with the local legislation and institutional requirements. Written informed consent to participate in this study was provided by the participants’ legal guardian/next of kin. Written informed consent was obtained from the individual(s), and minor(s)’ legal guardian/next of kin, for the publication of any potentially identifiable images or data included in this article.

## Author Contributions

PG: Design, writing, review of literature, report case, revision; DM: writing, review of literature; MK: review of literature, writing; EC: review of literature, revision; VV-G: review of literature, revision; AD: review of literature, report of case; FB: Design, writing, review of literature, report case, revision. All authors contributed to the article and approved the submitted version.

## Conflict of Interest

PG received honoraria from AbbVie and Novartis as a consultant and provided lectures for AbbVie, Brothier, Cicaplus, Coloplast, Inresa and Novartis.

The remaining authors declare that the research was conducted in the absence of any commercial or financial relationships that could be construed as a potential conflict of interest.

## Publisher’s Note

All claims expressed in this article are solely those of the authors and do not necessarily represent those of their affiliated organizations, or those of the publisher, the editors and the reviewers. Any product that may be evaluated in this article, or claim that may be made by its manufacturer, is not guaranteed or endorsed by the publisher.

## Supplementary Material

Supplementary Table 1 The predicted effects of the variant of MVK gene are summarized in the table.
