# Supplementary material for: Case Report: Comorbid Hyper-IgD Syndrome and Hidradenitis Suppurativa – A New Syndromic Form of HS? A Report of Two Cases
Source: Front Immunol. 2022 May 26;13:883811. doi: 10.3389/fimmu.2022.883811 (PMC9204359; doi:10.3389/fimmu.2022.883811)
Supplement: Supplementary Table 1 — The predicted effects of the variant of MVK gene are summarized in the table. [file Table_1.docx]

| **Variant Coordinate** | **Amino Acid Change** | **Structural Features of mutation site** | **Structural Damage** | **Structure – based Stability Predictions** |
| --- | --- | --- | --- | --- |
| NM_001114185.3:c.1165G>A | p.Val377Ile | The mutant residue is larger than the wild type residue. The mutated residue is located in a binding and activity domain. | No structural damage detected | ΔΔG mCSM: -0.746 kcal/mol (Destabilizing)  ΔΔG SDM: -0.590 kcal/mol (Destabilizing)  ΔΔG DUET: -0.575 kcal/mol (Destabilizing)  ΔΔG ENCoM: 0.162 kcal/mol (Destabilizing) |
| NM_001114185.3:c.612C>G | p.Asp204Glu | The residue is located in the active site. The mutant residue is larger than the wild type residue, and interferes with hydrogen bonding and a salt bridge formed between the wild type reside and lysine at position 13. | \|  \| **No structural damage detected** \| \| --- \| --- \| | ΔΔG mCSM: -0.794 kcal/mol (Destabilizing)  ΔΔG SDM: 1.060 kcal/mol (Stabilizing)  ΔΔG DUET: -0.158 kcal/mol (Destabilizing)  ΔΔG ENCoM: 0.067 kcal/mol (Destabilizing) |

**Table S1**
